# The Role of γδ T Cells in Ischemic Stroke: Insights From the Perspective of the Brain–Gut Axis

**DOI:** 10.1002/cns.71092

**Published:** 2026-08-18

**Authors:** Yilin Li, Yina Li, Yang Shen, Yikun Gao, Jin Wang, Zhihong Jian, Lijuan Gu

**Affiliations:** ^1^ Central Laboratory Renmin Hospital of Wuhan University Wuhan China; ^2^ Department of Anesthesiology Renmin Hospital of Wuhan University Wuhan Hubei Province China; ^3^ Department of Neurosurgery Renmin Hospital of Wuhan University Wuhan Hubei Province China

**Keywords:** γδ T cells, brain–gut axis, IL‐17, ischemic stroke, microbiota

## Abstract

**Background:**

Stroke is a leading cause of disability and mortality worldwide, with ischemic stroke accounting for approximately 87% of all cases. Despite advances in reperfusion therapy, effective treatments targeting secondary neuroinflammation remain limited. Accumulating evidence indicates that γδ T cells, particularly through the production of interleukin (IL)‐17, contribute to ischemic brain injury. As the intestine represents a major reservoir of γδ T cells, brain–gut communication may connect intestinal immune alterations with post‐stroke neuroinflammation. This review examines the role of γδ T cells in ischemic stroke from the perspective of the brain–gut axis.

**Methods:**

We synthesized current mechanistic evidence from experimental, translational, and clinical studies concerning γδ T‐cell activation and recruitment after ischemic stroke. Particular attention was given to IL‐17‐mediated neuroinflammation, intestinal barrier dysfunction, gut microbiota dysbiosis, and bidirectional immune communication between the ischemic brain and the intestine.

**Results:**

Following ischemic stroke, γδ T cells are activated and recruited to the injured brain, where their production of IL‐17 promotes neuroinflammation, blood–brain barrier disruption, and secondary tissue injury. Concurrently, cerebral ischemia induces autonomic, immune, and metabolic disturbances that alter the gut microbiota and compromise intestinal barrier integrity. These changes may facilitate microbial product translocation and systemic inflammation, thereby amplifying cerebral inflammatory responses. Gut‐resident γδ T cells may participate in this process by regulating mucosal immunity and IL‐17‐associated inflammatory signaling. Together, these mechanisms may establish a self‐reinforcing brain–gut injury cycle. However, direct evidence defining the contributions, trafficking patterns, and functional heterogeneity of specific intestinal γδ T‐cell subsets in ischemic brain injury remains limited.

**Conclusions:**

γδ T cells may serve as important immunological mediators linking intestinal dysfunction to neuroinflammation after ischemic stroke. Therapeutic strategies targeting γδ T‐cell activation, IL‐17 signaling, intestinal barrier disruption, or gut microbiota dysbiosis may help interrupt the brain–gut injury cycle. Future studies integrating lineage tracing, single‐cell and spatial profiling, and tissue‐specific interventions are required to distinguish the roles of brain‐infiltrating and gut‐resident γδ T‐cell subsets and facilitate the clinical translation of brain–gut axis‐based therapies.

## Graphical abstract

γδ T cells are pivotal in ischemic stroke, directly promoting brain inflammation and indirectly amplifying injury via the brain–gut axis. Through IL‐17 release, they disrupt the intestinal barrier, facilitating a harmful crosstalk that worsens stroke outcomes. Targeting this brain–gut immune axis presents a promising therapeutic strategy.

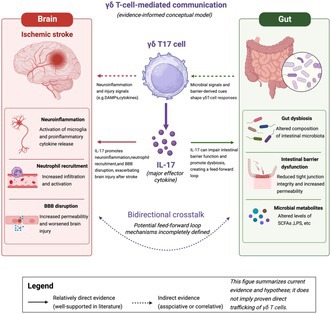

**Abbreviations**
BBB: blood–brain barrier
CNS: central nervous system
EA: electroacupuncture
EAE: experimental autoimmune encephalomyelitis
I/R: ischemia/reperfusion
IL: interleukin
TCR: T‐cell receptor
TLR: toll‐like receptors
TMAO: trimethylamine N‐oxide
Tregs: regulatory T cells
V: the variable regions

## 1 Introduction

Morbidity, disability, and mortality rates are still high for ischemic stroke patients, and the neurological deficiencies resulting from ischemic stroke are difficult to manage. Currently, most treatments affect symptoms but not the root cause of the disease. Because the pathogenesis of ischemic stroke is still being investigated, the efficacy of treatment for ischemic stroke is quite limited. Numerous studies have shown that the lymphocyte‐mediated inflammatory response is crucial in ischemic stroke. The inflammatory responses caused by cytokines secreted by lymphocytes after recruitment and activation critically exacerbate tissue damage and apoptotic cell death, thereby increasing the severity of stroke and accelerating the progression of cerebral ischemia/reperfusion (I/R) injury [1]. Among lymphoid cells, γδ T‐cell, which are members of the T‐cell family, and the cytokine IL‐17, which is mainly secreted by these cells, have gradually gained attention for their critical role in ischemic stroke. An increase in IL‐17 levels was observed on day three following stroke, according to transcellular cytokine staining. Injuries following I/R are mediated by IL‐17 and lead to apoptotic neuronal death at the infarct border zone. It was reported that reducing the number of infiltrating γδ T cells ameliorated the damage caused by I/R. γδ T lymphocytes seem to be potential targets for decreasing inflammation, which exacerbates injury following ischemic stroke [2].

γδ T cells are a specialized subset of T cells, and four peptide chains, α, β, γ, and δ, make up the T‐cell receptor. Compared with the more common and abundant αβ T cells, γδ T cells are fewer in number and have a heterodimerized surface T‐cell receptor (TCR) consisting of γ and δ chains. γδ T cells are mostly found in skin and mucosal tissues, such as the intestines, and have diverse immune functions. In addition to recognizing and killing cancer cells, the intrinsic immune system responds to infection and tissue damage through γδ T cells. Ischemic stroke is commonly linked to an inflammatory reaction, and γδ T cells can regulate this response. Multiple studies have shown that the body's defense against ischemic stroke relies heavily on γδ T lymphocytes [3, 4]. These cells become active following a stroke and travel to the location of brain damage. IL‐17, which is released from γδ T cells, is associated with poor ischemic stroke outcomes since these cells produce cytokines and other substances to improve tissue healing and regulate the immune system.

γδ T cells play a pivotal role during ischemic stroke; these cells are found mainly in mucosal and epithelial tissues in peripheral blood and peripheral organs, with the intestine having the highest concentration. Following ischemic stroke, intestinal immune changes may influence meningeal and cerebral γδ T‐cell responses, which may be related to the recently proposed brain–gut axis theory. An important function of the brain–gut axis is bidirectional communication between the central nervous system (CNS) and the enteric nervous system. The brain–gut axis involves complex interactions among the nervous system, the endocrine system, and the immune system. In recent years, much research and attention have been focused on this topic. Various physical and mental disorders, such as inflammatory bowel syndrome and depression, have been shown to be closely related to brain–gut axis dysregulation. Numerous studies have shown that the brain–gut axis plays a role in the pathophysiology of numerous neurological disorders, including ischemic stroke [4, 5]. The gut microbiota is also essential for maintaining interactions involved in brain and gut homeostasis, thereby affecting behavioral and cognitive functions in the CNS. Brain–gut communication may involve gut‐resident γδ T cells as one immune component. Recent evidence has shown that ischemic stroke and intestinal γδ T cells are more closely related than originally thought. Although γδ T cells and the brain–gut axis have been discussed separately in ischemic stroke, their mechanistic integration remains incompletely defined. The objective of this review is to further explore the role of γδ T‐cell in stroke from the perspective of the brain–gut axis.

## 2 Neurological Importance of γδ T Cells

### 2.1 Profile of the Main Characteristics of γδ T Cells

**FIGURE 1:**
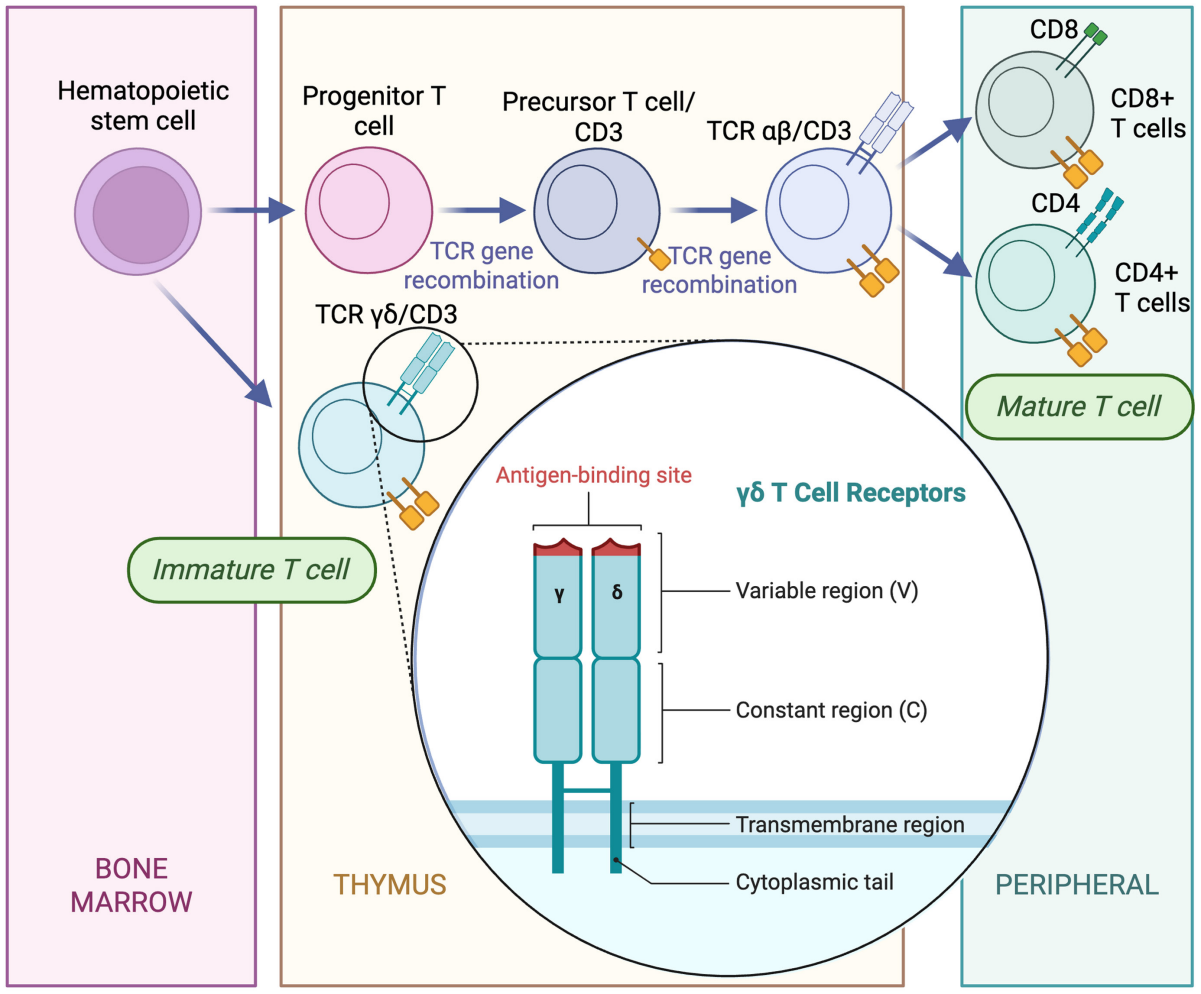
Bone marrow‐derived hematopoietic stem cells can develop in the thymus to form progenitor T cells, which undergo T‐cell receptor (TCR) δ, γ, β, and α gene rearrangements to form precursor T cells with the surface marker CD3, which are the most immature T‐cell precursors, and cells expressing the γδ TCR, which then differentiate into mature γδ T cells that migrate out of the thymus and move to the periphery under suitable conditions. In contrast, the subpopulation that undergoes positive selection is transformed into CD4 + CD8+ double‐positive cells and subsequently develops into αβ T cells, which in the periphery can differentiate into CD8+ T cells and CD4+ T cells. The fetal thymus is the site of the early development and acquisition of the effector function of γδ T cells, providing the first line of immune defense for the newborn.

γδ T cells are a subpopulation of T cells characterized by a TCR composed of a γ glycoprotein chain and a δ glycoprotein chain. Lymphoid and nonlymphoid tissues have different types of TCRs according to the types of connections between the Vγ chains and the Vδ chains of the variable regions (V) [6]. CD3 expression on the surface of γδ T cells is high, sometimes higher than that on αβ T cells, while CD4 and CD8 are not expressed (double‐negative) on γδ T cells. Occasionally, γδ T cells show partial expression of CD8. In humans and rodents, γδ T cells develop in the thymus and are prioritized over αβ T cells, but after birth, the percentage of γδ T cells, which are dominant in fetal life, decreases. Finally, γδ T cells make up only a small percentage of the T cells in peripheral blood and lymph nodes, approximately 1%–5% [7]. Some subgroups of γδ T cells released from the thymus are known to reside on mucosal and epithelial surfaces (Figure 1). Examples include the mouse Vγ5+ T‐cell subpopulation, which occupies a limited part of the intestinal epithelium. Considering that these barrier sites are home to a considerable number of γδ T cells, the microbiota or pathogens that invade these sites are usually able to influence them directly or indirectly. In many cases, γδ T cells act as early responders. When pathogenic microorganisms such as bacteria or fungi invade a cell, various cytokines are produced by multispecific γδ T cells to fight the infection early on and prevent the host from becoming further infected [8, 9, 10]. In addition to fighting infection, γδ T cells play crucial roles in cancer, autoimmune diseases, and tissue maintenance. Marta et al. recently reported that two major types of human γδ T cells, Vδ1 and Vδ2, differ in terms of phenotype, transcriptome, and functional response. In addition, further studies are needed to determine which ligands are recognized by each subset of γδ T cells, how their effector responses develop and how they interact with the immune system overall [11]. Following the elimination of pathogens or resolution of inflammation, certain γδ T cells establish enduring adaptive memory populations within the impacted tissues. Evidence suggests that both natural γδ T cells and γδ T cells partially induced after inflammation can have a positive impact, contributing to tissue balance and pathogen management. However, in specific situations, γδ T cells may also worsen local inflammation [12].

Importantly, γδ T cells are not a homogeneous population. Human Vδ1, Vδ2 and Vγ9Vδ2 subsets and mouse Vγ1, Vγ4, Vγ6 and Vγ7 subsets differ in tissue distribution and function. Mouse Vγ‐defined subsets cannot be directly equated with human Vδ‐defined subsets (Table 1).

**TABLE 1:** Major γδ T‐cell subsets relevant to the brain–gut axis.

Species/category	Subset	Main location	Functional tendency	Relevance to this review
Human	Vδ1	Tissue and mucosal sites	Barrier surveillance and local immune regulation	Potential relevance to intestinal immunity [11, 13, 14]
Human	Vδ2/Vγ9Vδ2	Peripheral blood	Rapid cytokine response and immune surveillance	Translational relevance for clinical studies [11, 14]
Mouse	Vγ1	Tissue‐dependent	Regulatory or protective potential in some models	May counterbalance inflammatory γδ subsets [15, 16]
Mouse	Vγ4	Inflamed tissues and CNS‐related models	IL‐17/IFN‐γ‐associated inflammation	Potential contributor to neuroinflammation [16, 17, 18]
Mouse	Vγ6/Vγ7	Tissue‐resident and intestinal epithelial compartments	Rapid IL‐17 response or barrier interaction	Relevant to gut‐resident γδ T‐cell biology [13, 14, 15]
Functional	γδT17	Gut, meninges and inflamed tissues	IL‐17A production	Most relevant to acute post‐stroke inflammation [1, 2]

### 2.2 Primary Functions of γδ T Cells via Secretion of IL‐17

Various cytokines, including IL‐17, IL‐21, IL‐22, and interferon‐γ, have been reported to be secreted by γδ T cells to facilitate inflammation and the immune response [19]. Our focus will be on IL‐17, a classic proinflammatory cytokine that plays a role in the adaptive immune system. IL‐17 can protect the body by inducing neutrophil recruitment, the production of antimicrobial peptides and the enhancement of barrier protection against invading fungi and bacteria, or it can lead to a wide range of diseases because of the imbalance in the inflammatory response that it causes [20, 21]. Therefore, γδ T cells should not be defined only by pathogenic IL‐17A production, because their functions vary according to subset, tissue context, and disease phase.

#### 2.2.1 Primary Effects of γδ T Cells in the CNS

As a key component of the immune response to neurological diseases, IL‐17, which is produced by γδ T cells distributed in the CNS, plays an important role in regulating inflammation [22, 23, 24]. Ribeiro et al. and Lima et al. demonstrated that meningeal γδ T cells regulate synaptic plasticity, short‐term memory, and anxiety‐like behaviors through IL‐17 signaling [25, 26]. Brain homeostasis and cognitive function are dependent upon γδ T cells [27]. Within a few days following cerebral microbleeds, γδ T cells accumulate and proliferate in the brain parenchyma, where they alleviate inflammation and support neuroprotection during the early stages of cerebral microbleeds [12, 28]. Cognitive function can be affected by a finely regulated balance of inflammatory cytokines secreted by γδ T cells [29]. Patients with Parkinson's disease have abundant γδ T cells in their peripheral blood circulation and cerebrospinal fluid, but the beneficial or deleterious effects of these cells on these patients remain unknown [30]. The number of IL‐17‐secreting γδ T cells in epileptogenic foci is correlated with epilepsy severity [31]. Mouse experimental autoimmune encephalomyelitis (EAE) is an animal model of multiple sclerosis, and γδ T cells are found in abundance in the brains of mice with EAE; EAE susceptibility is further increased by these large numbers of γδ T cells that promote the production of IL‐17 by CD4+ T cells [18, 32, 33]. γδ T cells with functionally distinct subpopulations have opposing roles in EAE; for example, when self‐antigens are present in the CNS, Vγ4+ cells, which predominate in EAE, further promote inflammation by producing proinflammatory cytokines such as IL‐17 [17]. Interferon‐γ secreted by activated γδ T cells activates the microglia‐A1 astrocyte‐C3 neurotoxic pathway, leading to neurodegenerative diseases such as amyotrophic lateral sclerosis and multiple sclerosis (Table 2) [34]. These findings suggest that γδ T cells can exert protective or detrimental CNS effects depending on subset identity, cytokine profile, anatomical location, and disease phase.

**TABLE 2:** Summary of the main mechanisms of action of γδ T cells in CNS diseases.

Diseases/pathological state	Species	γδ T subsets	Mechanisms	Conclusion	References
Short‐term memory deficiency	Mouse	—	Regulated by IL‐17 signaling	Beneficial	[16]
Anxiety‐like behavior	Mouse	—	Regulated by IL‐17 signaling	Detrimental	[17]
AD	Mouse	—	IL‐17 promotes early AD pathogenesis	Detrimental	[20]
PD	Human	—	The peripheral circulation and cerebrospinal fluid of PD patients contain many γδ T cells	Not yet confirmed	[21]
Refractory epilepsy	Human	—	The number of IL‐17‐secreting γδ T‐cell in epileptogenic foci is correlated with disease severity	Detrimental	[22]
EAE	Mouse	Vγ4	The promotion of IL‐17 production increases EAE susceptibility and tissue inflammation	Detrimental	[23, 24, 25, 26]
MS	Human	Vδ1/Vδ2	The interferon‐γ secreted by activated γδ T cells activates the microglia‐A1 astrocyte‐C3 neurotoxic pathway	Detrimental	[27]
ALS	Human	—	The interferon‐γ secreted by activated γδ T cells activates the microglia‐A1 astrocyte‐C3 neurotoxic pathway	Detrimental	[27]
CMB	Mouse	—	Aggregation and proliferation in the brain parenchyma to relieve inflammation and support neuroprotection in the early stages	Beneficial	[9, 19]

Abbreviations: AD, alzheimer's disease; ALS, amyotrophic lateral sclerosis; CMB, cerebral microbleed; CNS, central nervous system; EAE, experimental autoimmune encephalomyelitis; MS, multiple sclerosis; PD, parkinson's disease.

#### 2.2.2 Biological Functions of IL‐17+ γδ T Cells During Ischemic Stroke

In addition to the predominantly secreted Th17 cells, IL‐17 can also be produced by γδ T lymphocytes. IL‐17 plays a critical role in early ischemic stroke progression because of its inflammatory and destructive properties [2, 35, 36, 37, 38]. Functional γδ T cells are identified during intrathymic differentiation, and young γδ cells capable of producing IL‐17 express the transcription factor retinoic acid receptor‐associated orphan receptor γt and the specific cytokine receptor IL‐23R. γδ T cells produce IL‐17 as soon as they leave the thymus as a response to peripheral stimuli [32, 39]. For example, when ischemia occurs, IL‐23 (predominantly secreted by dendritic cells) can act synergistically with IL‐1β; in the presence of IL‐18 and chemokine receptor type 6 (CCR6), this synergy induces γδ T cells to produce IL‐17 and migrate to the affected area. Blood–brain barrier (BBB) damage is exacerbated by the released IL‐17 as it invades the injured site and attracts neutrophils, which also try to cross the damaged BBB [9, 40]. In one study, the infarct size was reduced, and neurological outcomes were improved as a result of IL‐23/IL‐17 cascade disruption [41]. The release of IL‐10 by regulatory T cells (Tregs), macrophages, and microglia limits the signaling of IL‐17A in deleterious γδ T cells, which stabilizes the neuron during an ischemic stroke [42, 43]. In contrast, signaling by IL‐1, a classic proinflammatory cytokine, mediates neutrophil recruitment targeting γδ T cells, which can be induced in vivo to produce IL‐17A to exacerbate early inflammation after ischemic stroke [1, 44]. Signaling pathways involving toll‐like receptors (TLR) facilitate CNS damage. In vitro co‐culture experiments have demonstrated that TLR‐activated microglia can drive γδ T‐cell polarization towards an IL‐17‐producing neurotoxic phenotype. Importantly, this neurotoxicity was shown to depend on direct cell–cell contact between γδ T cells and neurons, suggesting a potential role for membrane‐bound effector molecules. Further in vivo validation is needed to confirm whether this contact‐dependent mechanism operates in physiological contexts [45, 46]. These findings illustrate that γδ T cells play important roles in neuronal function, the immune‐inflammatory response and the pathology of the CNS. Determining the role of γδ T cells in the CNS can provide insights into the pathogenesis of neuroinflammatory diseases such as ischemic stroke and potential therapeutic strategies. Taken together, these findings suggest that the detrimental effect of γδ T cells in ischemic stroke is mainly associated with IL‐17A‐producing γδT17 cells rather than all γδ T‐cell populations. The γδT17–IL‐17A axis may be most relevant during the acute and early subacute phases, whereas the role of γδ T cells during later recovery remains unclear. Most mechanistic evidence remains preclinical, and human subset‐specific evidence is still limited.

## 3 Functions of γδ T Cells in Ischemic Stroke Occurring Through the Gut

### 3.1 γδ T Cells in the Intestine: Roles and Functions

There is a high concentration of γδ T cells in the intestinal mucosa, particularly in epithelial tissues, despite being sparsely distributed in circulating blood and lymphoid tissues. These cells have a significant effect on tissue balance, maintaining barrier strength, repairing damage, and affecting mucosal health and disease. A significant number of CD8+ cells are found among intestinal intraepithelial lymphocytes, with IL‐2R signaling being crucial for the formation of γδ intestinal intraepithelial lymphocytes [47, 48]. The mammalian intestinal mucosa has evolved into an unexpected immune region to protect itself against pathogenic attacks. As parts of the body's innate immune system, intestinal γδ T cells segregate pathogenic microorganisms by rapidly producing cytokines and growth factors to prevent their spread. When neutrophils are recruited and phagocytes and dendritic cells are activated, the immune system produces an early adaptive response.

There is also an interaction between the gut microbiota and γδ T cells that affects the development of diseases and tissue homeostasis. Through the brain–gut axis, IL‐17 produced by γδ T cells regulates intestinal inflammation and defends the host in response to the microbiota [49]. Histone deacetylase‐dependent inhibition of the production of IL‐22 and IL‐17 by cecal γδ T cells was observed when gut microbiota derivatives, such as propionic acid, were administered [50]. Gut microbiota composition or structure can also cause inflammation of the CNS. For example, it was found that *Lactobacillus acidophilus* stimulates the development of Vγ1+ γδ T cells with inhibitory capacity for the CNS, and these γδ T cells are denoted as regulatory γδ T cells, but Vγ4+ γδ T‐cell development was hindered [16]. Inflammatory conditions, such as hypoxia, activate intestinal γδ T cell production of angiopoietin‐4, which regulates gut microbial composition. The abundance of *Clostridium difficile* was found to be significantly reduced in the mouse intestine, whereas the abundance of *Vibrio desulfuricans* was significantly elevated. The phosphatidylethanolamine and phosphatidylcholine derived from *Vibrio desulfuricans* promote γδ T‐cell activation, resulting in intestinal damage [51]. Studies have shown that even though vagal and microbial metabolites fundamentally regulate gut ecology over the long term, microbiota‐derived and neuroimmune signals may influence nervous system function through multiple immune pathways, including γδ T‐cell‐related responses [9]. This evidence also suggests that intestinal γδ T‐cell responses should be interpreted in a subset‐specific manner. For example, regulatory‐like Vγ1+ γδ T cells and inflammatory Vγ4+ γδ T cells may have different effects. These findings should not be interpreted as direct proof that a defined intestinal γδ T‐cell subset migrates to ischemic brain tissue.

### 3.2 A Critical Role for the Brain–Gut Axis and Gut Microbiota in Ischemic Stroke

**FIGURE 2:**
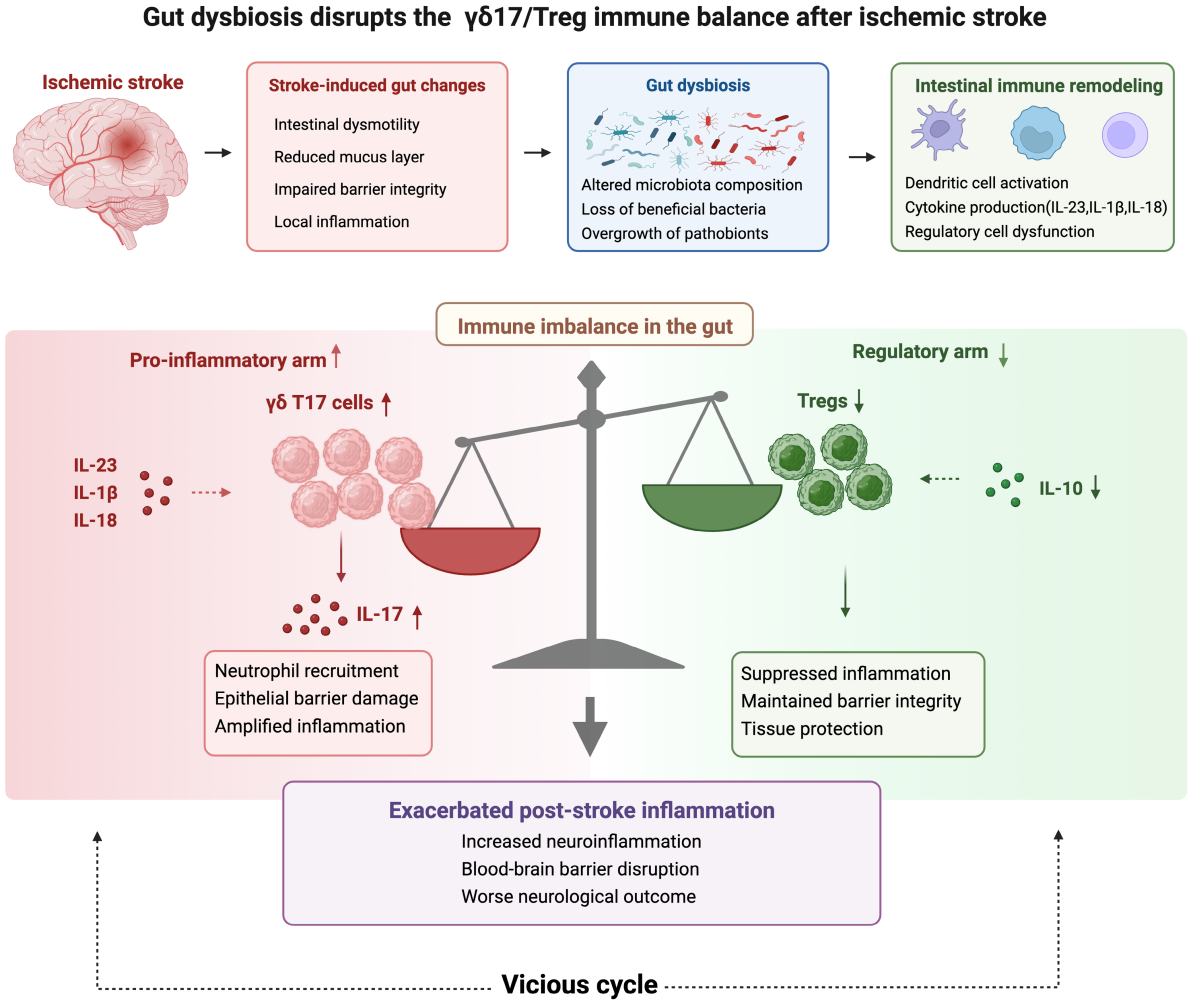
The protective immune response to interleukin (IL)‐17 is usually controlled by Tregs and their secretion of the anti‐inflammatory cytokine IL‐10, which, if unbalanced, promotes immunopathology in the context of autoimmune or inflammatory diseases or further contributes to the development of diseases such as ischemic stroke. After the onset of ischemic stroke, factors such as gut microbiota dysfunction, neurological disorders, increased permeability, and gut immune cell activation can be affected, which in turn leads to increased secretion of proinflammatory cells or bacterial toxins; for example, the levels of IL‐17 secreted by γδ T cells, which are distributed in the gut epithelium and mucosal tissues, can increase, exceeding the limitations of Tregs, which can then leach through the compromised blood–brain barrier into brain tissues and ultimately aggravate ischemic stroke.

Brain and gut activity are integrated in the host through a bidirectional neurohormonal communication system called the brain–gut axis [52, 53, 54, 55]. Through the brain–gut axis, the gut microbiota modulates neuronal function and behavior by producing metabolites such as neuroactive compounds. The effects of this can be observed in the host's metabolic and immune system activity, which in turn affects gut neuronal pathways [56, 57]. Ischemic stroke may disrupt the brain–gut axis by leading to gut microbiota dysfunction, neurological disturbances, increased intestinal permeability, and intestinal immune cell activation. As a result, proinflammatory cells or bacterial toxins penetrate the damaged BBB, causing cerebral infarctions to worsen (Figure 2) [58]. Even further, Carolina et al. novelly suggested the existence of a microbiota–gut–inflammatory vesicle–brain axis, where intestinal bacteria can modulate the inflammatory response through inflammatory vesicle NLRP3 signaling, thereby affecting brain homeostasis [59]. Studies in related neurological conditions also support a broader interaction among gut microbiota, IL‐17‐producing γδ T cells, and neuroimmune signaling, providing additional context for understanding gut–immune–brain communication after ischemic stroke [60, 61]. Overall, these findings suggest that communication between the brain and intestine involves neural, immune, microbial, and metabolic pathways.

Intestinal microbiota composition and function affect the organism through a variety of mechanisms. Patients with ischemic stroke have a different gut microbiota composition than those without ischemic stroke. In patients, there was an increase in the relative abundance of opportunistic pathogens, whereas there was a decrease in the relative abundance of commensal bacteria [62]. The effects of ischemic stroke can be mitigated by modulating gut commensal microbes through multiple methods, including antibiotic administration [4]. In a study by Savigamin et al., probiotics ameliorated neurological deficits and had anti‐inflammatory and antioxidant properties, which reduced brain volume loss and inhibited neuronal apoptosis [63]. Transplanting the gut microbiota from young mice to older mice has been shown to improve stroke outcomes [64]. Several types of gut bacterial traits may increase or decrease the risk of ischemic stroke, according to Meng et al. Furthermore, different specific bacteria are associated with varying degrees of risk for different types of ischemic stroke [65, 66]. A substance called trimethylamine N‐oxide (TMAO) is produced by gut microorganisms that metabolize choline and act on the CNS via the brain–gut axis [67]. TMAO modulates cholesterol metabolism, platelet reactivity and thrombosis, as well as inflammation and oxidative stress, which are significant contributors to the onset, development, and prognosis of ischemic stroke [68, 69, 70]. Hence, new treatments and predictions for ischemic stroke may benefit from targeting the gut microbiota and its metabolites [71].

### 3.3 Brain–Gut Interactions in Ischemic Stroke

The dysregulation of the gut microbial ecology that results from ischemic stroke is associated with metabolic disorders and the body's inflammatory response. An increase in the number of IL‐17A+ γδ T cells in ischemic brain tissue can increase intestinal permeability, alter the microbial composition of the intestinal mucosa, and activate the intestinal immune system [72]. Infection of the brain occurs when the BBB is compromised, allowing ectopic intestinal bacteria and proinflammatory cells to enter [73, 74, 75]. Ischemic stroke reduces gut levels of short‐chain fatty acids, which support a healthy gut microbiota by inhibiting the growth of harmful bacteria [76, 77]. In addition to affecting gut microbes and their metabolites, ischemic stroke damages the intestinal epithelium and activates the gut immune response [78, 79]. Intestinal motility disorders are often associated with damage‐associated molecular patterns that trigger innate and adaptive immune responses via specialized pattern recognition receptors (e.g., TLR) in ischemic brain tissue. As mentioned earlier, TLR‐activated microglia play crucial roles in polarizing neurotoxic IL‐17+ γδ T cells. In summary, ischemic stroke may influence gut immune responses, and γδ T cells may represent one immune link between the gut and the brain.

Intestinal γδ T cells may act on distant organs indirectly, such as the brain, in response to psychological stress and ischemic stroke [3]. Stress causes changes in the gut microbiota, resulting in the migration of γδ T cells into the meninges [80]. Social isolation‐induced ischemic brain injury is ameliorated by recombinant human atrial natriuretic peptide, which inhibits the migration of small bowel‐derived γδ T cells to the brain [81]. Alterations in the gut microbiota after stroke, along with harmful metabolites (e.g., branched‐chain amino acids) that promote IL‐17 secretion by γδ T cells, can further damage the poststroke brain via gut IL‐17+ γδ T cells. Additionally, poststroke gut microbiota—particularly Lactobacillus species—produce metabolites that directly suppress γδ T cell‐mediated neuroinflammation (e.g., by reducing IL‐17 production) and independently promote neurological repair [82]. For example, after colonization, *Lactobacillus helveticus* and *Lactobacillus brevis* showed significant neuroprotective activity and greatly attenuated the accumulation of branched‐chain amino acids, (which drive γδ T cell activation) in both ischemic stroke patients and I/R rats [83]. Singh et al. reported that bacterial colonization reduces the incidence of ischemic stroke in germ‐free mice [84]. Interestingly, microbial‐mediated brain protection was absent in lymphocyte‐deficient mice, suggesting a role for lymphocytes—likely γδ T cells—in mediating this effect. To confirm the concept of protective neuroinflammation driven by lymphocytes, particularly γδ T lymphocytes, under microbial control after stroke, the imbalance of the inflammatory response to further damage caused by stroke should be explored in future studies, and how to define the threshold of the imbalance and anticipate this threshold to provide optimized treatment to stroke patients is a promising yet rewarding path of research [53, 84]. Current evidence supports a relationship among gut microbiota, intestinal immune remodeling, γδT17 responses, and ischemic stroke outcome. However, direct proof that specific gut‐derived γδ T‐cell subsets migrate into ischemic brain tissue and determine injury severity remains insufficient. Future studies using lineage tracing, single‐cell RNA sequencing, TCR sequencing, and spatial validation are needed.

## 4 Therapeutic Approaches to Ischemic Stroke Derived From Gut γδ T Cells

Studies on the mechanisms of action of traditional Chinese medicine have shown that γδ T‐cell‐related pathways may represent potential therapeutic targets in ischemic stroke. The Chinese medicine Naochang Tongtiao acupuncture has been shown to ameliorate neurological function in ischemic stroke patients, and its effectiveness is superior to that of conventional acupuncture. The serum levels of IL‐17 and C‐reactive protein decrease, and plasma TMAO levels decrease after treatment [85]. Moreover, the treatment of cerebrovascular diseases with electroacupuncture (EA) is common [86]. Ischemic stroke rats exhibited a reduced infarct volume and intestinal barrier damage following EA. Rats with I/R‐induced injury responded well to this therapy, which suppressed the brain and small intestinal inflammation that follows ischemic stroke, ameliorating intestinal inflammatory injury and improving intestinal barrier integrity [87]. A further contributing factor to the protective effect of EA following ischemic stroke is the inhibition of intestinal T‐cell mobilization by EA [88]. Herbal medicines such as hypericin alleviate inflammation by modulating the MAPK/STAT3 pathway and inhibiting the immune response of IL‐17+ γδ T cells and related cytokines [89]. These findings suggest that gut‐directed or immune‐modulating interventions may influence post‐stroke immune responses, potentially involving γδ T‐cell‐related pathways. However, whether their protective effects depend on specific γδ T‐cell subsets or brain‐to‐gut trafficking routes requires further validation.

## 5 Conclusions and Expectations

**FIGURE 3:**
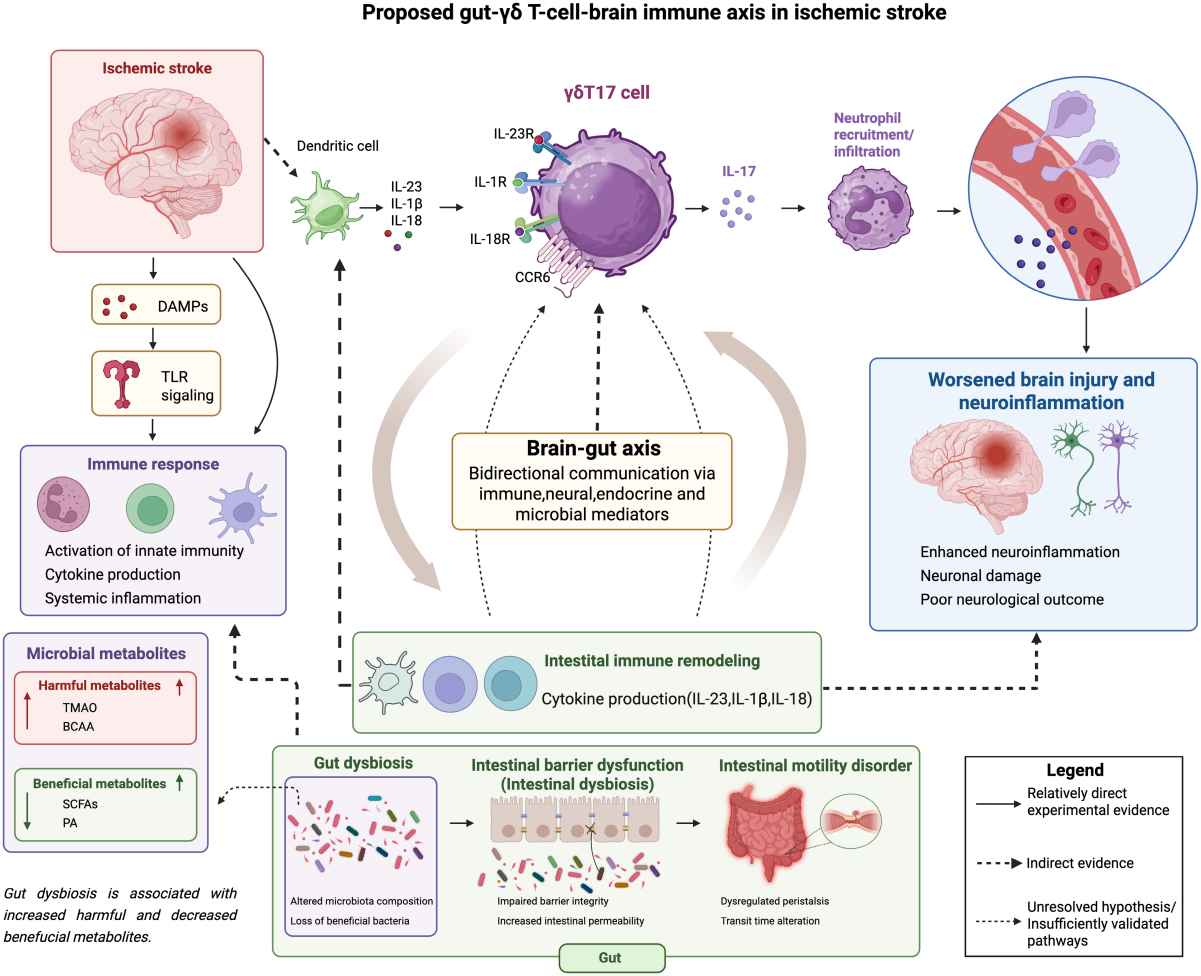
Overview of γδ T cells as pivots in bidirectional brain–gut communication during ischemic stroke. BBB, blood–brain barrier; BCAA, branched‐chain amino acid; CCR6, chemokine receptor type 6; DAMPs, damage associated molecular patterns; IL, interleukin; PA, propionic acid; SCFA, short‐chain fatty acid; TLR, toll‐like receptor; TMAO, trimethylamine N‐oxide.

γδ T cells are integral components of the immune system that contribute to host defense and inflammatory regulation. The brain–gut axis is a complex neuroendocrine‐immune network that has recently been linked to neurological diseases, including ischemic stroke. Upon ischemic stroke onset, γδ T cells may be recruited to damaged brain tissue and become activated. These cells may help detect and clear damaged cells and pathogens, thereby limiting secondary injury. The overactivation of γδ T cells can, however, further aggravate brain damage by provoking an increased inflammatory response. In addition to their direct role in the immune response, γδ T cells can also affect ischemic stroke through the brain–gut axis. The immune and inflammatory responses induced through γδ T cells after ischemic stroke can also exacerbate the disruption of the intestinal microbial environment, resulting in gut injury and leading to the development of a vicious cycle; thus, we propose the “brain–γδ T cell–gut” loop (Figure 3) as an evidence‐informed conceptual model rather than a fully established linear pathway. Accordingly, the effect of γδ T‐cell on ischemic stroke through the brain–gut axis is an intriguing and complex research topic. A new perspective on ischemic stroke pathogenesis has been proposed, as have new ideas for preventing and treating ischemic stroke.

The present knowledge of γδ T lymphocyte function in ischemic stroke is encouraging. However, there are several limitations to this understanding. A better understanding of how stroke‐related γδ T‐cell dysfunction regulates brain–gut axis function and the gut microbiota is still lacking. According to the literature, γδ T‐cell may play a significant role in ischemic stroke pathophysiology and recovery, but further work is needed to elucidate how these cells interact with the gut after ischemic stroke. To determine whether targeting these cells can offer therapeutic benefits, more detailed pathways along the brain–gut axis need to be explored. Studies on the role of γδ T cells in ischemic stroke in the literature have primarily been conducted in mouse models. Clinical research and human trials are lacking. In addition to γδ T cells, there are several subtypes of them, with different functions depending on where they are located and when they are activated. There are no clear definitions of the specific subtypes of γδ T cells associated with ischemic stroke or their functions. Further investigations are warranted regarding the timing and duration of γδ T‐cell activation during the ischemic stroke response and its relationship with ischemic stroke progression and recovery. Future studies should also consider subset‐specific and stage‐dependent roles of γδ T cells, including differences between inflammatory γδT17 cells and potentially protective or regulatory γδ T‐cell populations. Ischemic stroke is characterized by a complex immune response involving a variety of cell types and pathways. There is uncertainty about the precise role that γδ T cells play in this response and how they interact with other immune cells. The manipulation of γδ T cells is promising for ischemic stroke treatment, but the long‐term effects are not known. Overall, current findings support further investigation of γδ T‐cell function under ischemic stroke conditions, but much more research is needed before this information can be used to develop effective ischemic stroke treatments. Further research is required to understand the role and mechanisms of γδ T cells in ischemic stroke pathogenesis and progression, including their impact on the brain–gut axis, to mitigate neuroinflammation and enhance neurological recovery post‐stroke.

## Author Contributions

**Yilin Li:** wrote the initial draft. **Yina Li:** contributed to reviewing the literature. **Yang Shen** and **Yikun Gao:** helped to prepare the manuscript. **Jin Wang:** prepared the figures and submitted the article. **Lijuan Gu** and **Zhihong Jian:** designed the manuscript and prepared the final version.

## Funding

This study was supported by the National Natural Science Foundation of China (Nos. 82471370 and 82271370) to Lijuan Gu.

## Disclosure

We declare that we have not used any artificial intelligence content generation tools to write the manuscript.

## Conflicts of Interest

The authors declare no conflicts of interest.

## Data Availability

Data sharing not applicable to this article as no datasets were generated or analysed during the current study.
